# Stressors and Coping Strategies among Nursing Students during the COVID-19 Pandemic: Scoping Review

**DOI:** 10.3390/nursrep11020042

**Published:** 2021-06-03

**Authors:** Aisha Majrashi, Asmaa Khalil, Elham Al Nagshabandi, Abdulrahman Majrashi

**Affiliations:** 1 Medical-Surgical Nursing Department, King Abdulaziz University, Jeddah 21589, Saudi Arabia; 2 Faculty of Nursing, King Abdulaziz University, Jeddah 21589, Saudi Arabia; akhleel@kau.edu.sa (A.K.); elham@kau.edu.sa (E.A.N.); 3 Faculty of Nursing, Ain Shams University, Cairo 11566, Egypt; 4 Critical Care Medicine, Queen Mary University of London, London E14NS, UK; emsabood@gmail.com

**Keywords:** coping strategies, COVID-19 pandemic, nursing students, stressors

## Abstract

COVID-19 has impacted every aspect of life around the world. Nursing education has moved classes online. Undoubtedly, the period has been stressful for nursing students. The scoping review aimed to explore the relevant evidence related to stressors and coping strategies among nursing students during the COVID-19 pandemic. The scoping review methodology was used to map the relevant evidence and synthesize the findings by framing the research question using PICOT, determining the keywords, eligibility criteria, searching the CINAHL, MEDLINE, and PubMed databases for the relevant studies. The review further involved study selection based on the PRISMA flow diagram, charting the data, collecting, and summarizing the findings. The critical analysis of findings from the 13 journal articles showed that the COVID-19 period has been stressful for nursing students with classes moving online. The nursing students feared the COVID-19 virus along with experiencing anxiety and stressful situations due to distance learning, clinical training, assignments, and educational workloads. Nursing students applied coping strategies of seeking information and consultation, staying optimistic, and transference. The pandemic affected the psychological health of learners as they adjusted to the new learning structure. Future studies should deliberate on mental issues and solutions facing nursing students during the COVID-19 pandemic.

## 1. Introduction

The coronavirus disease 2019 (COVID-19) pandemic is the latest global health disaster of the century with high morbidity and mortality rates. In December 2019, a new infectious respiratory disease appeared in Wuhan, China. The World Health Organization (WHO) named the disease “COVID-19” after confirming its pandemic level potential. According to WHO, on 18 April 2020, the current outbreak of COVID-19 had affected over 2,164,111 people, and more than 146,198 deaths had been confirmed in more than 200 countries worldwide [1]. COVID-19 is one of the fastest spreading viral infections, which WHO declared a pandemic after affirming the high infectious levels. The disease spread from person to person through infected air droplets released through coughing or sneezing. Additionally, people spread the virus through physical contact, such as greetings or touching infected surfaces. Countries have sought vaccines and treatment protocols for COVID-19 vaccines and treatments amidst the implementation of various containment measures worldwide to combat the disease. Such containment measures have included the closure of public places, schools, universities, imposing curfews, and other physical distancing measures, such as the cancelation of large events [2].

The COVID-19 pandemic has forced political leaders and universities to take drastic measures to safeguard citizens’ and students’ lives. As many universities have suspended classroom teaching and switched to online teaching, the lives of students have changed completely and students have become prone to developing stressors, such as fear about physical health, family, and a loss of control related to the change in the educational environment [3].

Stress refers to a “situation in which internal demands, external demands, or both, are appraised as taxing or exceeding the adaptive or coping resources of an individual or group” [4]. Nursing students can suffer from a high level of stress during their education program. Specifically, there are two significant sources of stress among nursing students, academic and clinical stressors. The stressors related to academia include heavy assignments, examinations, and workloads. Other sources of stress related to the clinical area for nursing students include a lack of professional nursing skills and unfamiliarity with patients’ diagnoses, medical history, or treatment [4].

The specific stressors related to the impact of COVID-19 among nursing students are stress from COVID-19 infection and a lack of preventive measures in clinical training [1]. This period has been undoubtedly stressful for learners; with classes moving online, nursing students face difficulties, such as being unable to concentrate and having difficulties participating, writing assignments, taking exams, and meeting the deadlines of academic assignments [5].

Coping strategies are stabilizing methods for helping individuals maintain psychological adaption during stressful events [6]. Coping strategies are classified as problem-based or emotion-based coping. The problem-solving approach is the most common coping strategy employed by nursing students to adjust to stressors, while an avoidance approach is the coping behavior least used by nursing students [4]. Nursing students use strong resilience, as one of the coping strategies during COVID-19 has been strong resilience. The learners have used humor, which studies associate with lower to moderate anxiety levels. Additionally, other coping strategies, such as mental disengagement, have led to high levels of anxiety [7]. This study sought to unearth the specific stressors and coping strategies employed by nursing students in universities during the COVID-19 pandemic.

### Aim of the Study

This scoping review aimed to explore the relevant evidence related to stressors and coping strategies among nursing students during the COVID-19 pandemic.

## 2. Materials and Methods

A scoping review methodology was used to map the relevant evidence and synthesize the findings. The 6 steps of scoping review by Pérez et al. [8] guided the study. The steps involved the identification of the research question, determining the keywords, inclusion and exclusion criteria, searching the databases for the relevant studies, the study selection, charting the data, collecting, and summarizing the findings.

### 2.1. Research Question Formulation Using PICOT

The PICOT framework was used to develop and frame the research question “What are the stressors and coping strategies during the COVID-19 pandemic among nursing students?” The PICOT Framework in Table 1 generated the keywords used to undertake the research process in the selected electronic databases.

### 2.2. Key Words

A combination of the following terms was used to search the databases, using Boolean operators (“and”, “or”): “Nursing students”, “COVID-19”, “coronavirus”, “stressors”, “coping strategies”, “pandemic”, and “outbreak”.

### 2.3. Inclusion Criteria

The inclusion criteria for the searched articles were full-text articles in the English language from 2010 to 2020. The review further included studies that addressed stressors and coping strategies among students in line with the research question. Studies with quantitative methods were included in the review. Peer-reviewed and scholarly articles published within 5 years between 2016 and 2021 were included in the study.

### 2.4. Exclusion Criteria

The exclusion criteria for the articles included letters, reports, conference abstracts, dissertations, book chapters, and unpublished manuscripts.

### 2.5. Search Strategies

Various electronic databases, including CINAHL, MEDLINE, and PubMed, were searched using the pre-determined keywords to find the relevant articles that explore stressors and coping strategies among nursing students during the COVID-19 pandemic. After searching in the 3 databases, 15 articles were found in CINAHL, 20 articles in MEDLINE, and 24 articles in PubMed. Other databases utilized in this process generated 24 studies. Out of the 24 articles, 13 were from Google Scholar, 6 from JSTOR, 3 from ERIC, and 2 from Gale. In addition, Google Scholar was also used to locate open access articles. The keywords used in the search process include “Nursing students”, “COVID-19”, “stressors”, “coping strategies”, and “pandemic and outbreak”. The Boolean operators “AND” and “OR” were used to combine the keywords to create a focused search in each database.

### 2.6. Study Selection Process

The search strategy on the electronic database generated many articles. Inclusion and exclusion criteria were developed to guide the study selection and screening process. The inclusion criteria comprised articles that were full-text articles published in the English language from 2010 to 2020. The articles used quantitative research designs and journals that addressed the stressors as well as coping strategies during the COVID-19 pandemic among the nursing students. The exclusion criteria featured articles in the form of letters, reports, conference abstracts, dissertations, and unpublished manuscripts. The selection excluded articles that failed to address the concept of stressors and coping mechanisms among nursing students during the COVID-19 pandemic. Journals that lacked quantitative research designs, or were published in other languages besides English before 2010, were excluded in the final count.

**Figure 1:**
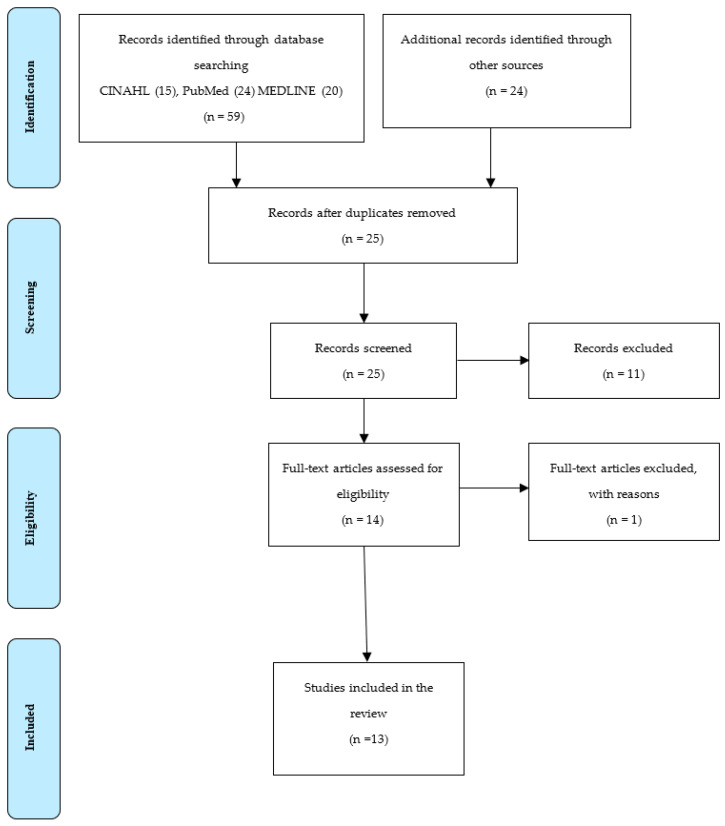
PRISMA flow diagram.

The PRISMA flow diagram guided the process of retrieval and the screening of studies (see Figure 1) after the most relevant articles were identified through the search process. In the article retrieval and screening process, a total of 83 studies were initially retrieved. Out of these articles, the screening process commenced, and 58 articles were removed due to duplication. The remaining 25 studies were subjected to the full-text examination to check their objective and relevance to the research question and a further 11 were excluded, and 1 article was removed.

### 2.7. Quality Assessment

The 13 studies that meet the eligibility criteria of the scoping review were assessed using separately using Hawker’s Quality assessment tool. The quality assessment tool evaluates the abstract, title, introduction, aims, methods, data, sampling procedure, data analysis, ethics and bias, as well as findings and results [9]. The assessment further focuses on the transferability, different implications, and usefulness of each study using a 36-points scale where each point received a maximum of 4 points ranging from 1 to 4 to signify poor and good quality respectively. The overall quality grades we used the following definitions were good (4), fair (3), poor (2), and very poor (1). Table 2 shows the quality appraisal for the studies in this review.

### 2.8. Charting of the Data

This phase comprised the extraction of the appropriate data from the selected literature to compile important insights to answer the research question, as recommended [21,22]. The extraction of the appropriate data assisted in identifying the relevant variables for answering the primary review question. According to [8], the data extraction process reduces bias and improves the overall reliability and validity of the review. The recorded information comprised the characteristics of the study, such as the authors, the sample size and setting, the country of study, the study design, the measurement tools, and the quality assessment. A thematic framework guided the presentation of the narrative accounts of the 13 studies, which then initiated the collection, summaries, and descriptions of the main findings (see Table 3).

## 3. Results

### 3.1. Results of the Search

This section outlines the evidence gathered from the selected literature for the review. It highlights the characteristics of the review study, the key results, and themes arising from the thirteen journal articles.

### 3.2. Characteristics of the Reviewed Studies

All the studies met the inclusion criteria and were published between 2010 and 2021. The review of the studies revealed the deployment of three key research designs of cross-sectional studies [7,10,11,13,14,15,18,19,20], mixed-methods [12] observational prospective studies [17], descriptive designs [5], and qualitative designs [16]. The sample comprised nursing exclusively, which fostered the generalizability to other nursing studies. Furthermore, the reviewed findings were from different geographical locations, including developing and advanced healthcare systems. The countries included Australia, India, Turkey, Egypt, Saudi Arabia, Spain, the United States, Israel, Croatia, and Jordan. Table 2 outlines the measurements and sample sizes gathered in each study.

### 3.3. Main Findings

Thirteen articles were selected and subjected to thematic analysis, showing different themes. The discussion used seven themes based on the information retrieved from them. Those seven themes fall into the broad categories of stressors and coping strategies. The first four themes are related to the nursing students’ stressors, while the last three are related to the coping strategies employed by those students.

#### 3.3.1. Theme 1: Nursing Students’ Stressors

Nine articles were concerned with studies on COVID-19-related stressors, from which four subthemes emerged. The first subtheme is “stress from distance learning”, in which issues such as remote learning’s psychological impacts are covered. The second subtheme is “stress from assignments and workload”. The third subtheme is “stress from clinical training”. The fourth subtheme is “fear of infection”, and it covers issues such as feeling isolated and worrying about getting infected.

##### Stress from Distance Learning

Eight articles provided a link between the COVID-19 pandemic and nursing students’ stress, with a focus on various stressors, including distance learning. During the COVID-19 pandemic, distance learning is a significant source of stress for nursing students. A cross-sectional study was conducted in 13 different nursing colleges in Nepal on 1116 participants [18]. The study aimed to assess the impact of E-learning during the COVID-19 pandemic among nursing students. The study found that many students suffered from the disruption of online classes related to the technological issues that occurred since higher learning institutions had moved to online classes. More than 63.2% of nursing students suffered from electricity problems, while 63.6% of nursing students suffered from internet problems, and only 64.4% of nursing students had internet access in the home for their online classes. Another cross-sectional study by [7] in Israel on 244 nursing students at Ashkelon Academic College assessed the levels of anxiety and coping strategies among nursing students during the COVID-19 pandemic. The study discovered that nursing students suffered significantly high levels of anxiety due to challenges presented by distance learning.

A qualitative study by [16] on 33 nursing students in Croatia explored how nursing students perceived the COVID-19 pandemic and their studying experience during the period. Distance learning presented many challenges to nursing students, including difficulties concentrating, as opposed to what they would do in a typical lecture room or face-to-face environment. The study also noted that nursing students found it difficult to remember and develop the motivation to undertake distance learning.

An observational and prospective study by [17] was on 142 nursing students in their second year in Murcia, Spain. The study’s purpose was to assess the levels of stress among nursing students before and during the COVID-19 lockdown and its influence on taking online exams. The study established that levels of stress significantly increased among nursing students after lockdown. In addition, the study noted that the students who failed the online exam had higher levels of stress compared to those who passed.

The perceived stress levels and poor concentration emerged in another cross-sectional study by [19]. The study on 662 nursing students in Turkey evaluated nursing students’ views on the COVID-19 pandemic and their perceived stress levels. The study proved that the nursing students suffered from moderate stress levels, but they had higher levels of stress than students assessed in the previous year. Nursing students expressed concerns about their clinical practice and inadequate clinical skills related to the interruption of education and moving to online learning during the pandemic.

Another cross-sectional descriptive study by [10] recruited 184 nursing students from universities in Nepal. The study assessed the factors associated with perceived stress, anxiety, and insomnia during the COVID-19 pandemic among nursing students. The study showed that 29.9% of the nursing students were afraid of delayed graduation, 36.4% suffered from costly mobile data and the necessity of spent money on mobile charging devices for their online classes, 17.4% had difficulties attending online access, 29.3% had difficulties concentrating, and 15.2% they were afraid of failure because they were unable to understand the online classes. The findings were confirmed in another study by [11], where they undertook a cross-sectional descriptive study on 244 nursing students in India. The authors assessed the perceived stress among nursing students during the COVID-19 lockdown. The study established that nursing students had moderate levels of stress related to a lack of resources and distance learning challenges.

Moreover, in a mixed-methods study by [12] that was conducted in Jordan on 335 nursing students, they analyzed the stress levels and sources of stressors related to distance learning and experienced by nursing students during the COVID-19 pandemic. It became evident that overall stress levels were higher among nursing students with low family income; 84.2% of the participants had a financial burden from paying for internet services. Furthermore, the Jordanian study found that distance learning has presented many stressors to nursing students, including difficulties concentrating because of distracting environments and no private areas for studying, limited resources, unorganized workloads, and a lack of strategies for standardized distance learning.

##### Stress from Assignments and Workload

Three articles reported that during the COVID-19 pandemic, assignments and workloads were a vital stress source for nursing students. One of these was the study conducted by [10], which reported that the global pandemic has affected university students in many ways. For nursing students working in a hospital, 44.4% suffered from long hours of duty, and 16.7% experienced increased workloads related to increased numbers of patients infected with the COVID-19 virus. Correspondingly, a study conducted by [7] reported that, in relation to the increase in the number of cases of the COVID-19 virus, there was a need to hire nursing students due to labor shortages in hospitals and in the community during the pandemic. Approximately 69% of the nursing students employed by hospitals had increased levels of anxiety. According to a descriptive study by [5], conducted in the United States among nursing students, learners showed difficulties in handling assignments, too. The study found that of the 84% of nursing students feeling anxious and overwhelmed, 62% had difficulty handling the academic workload, while 20% of nursing students had stress and difficulty writing assignments.

##### Stress from Clinical Training

Moreover, four articles found that stress from clinical training is one of the stressors that affected nursing students from around the world during the COVID-19 pandemic. In the first article by [7], which was conducted in Israel at Ashkelon Academic College, 50% of participants reported that they suffered from a lack of personal protective equipment (PPE) at the workplace. According to the study among nursing students, a lack of PPE was associated with higher anxiety scores in comparison with those students who did not suffer from a lack of PPE at the workplace.

Another study by [19] found that the main stressors for student nurses during this pandemic include adhering to COVID-19 precautions due to lack of adequate preparation. The third study was conducted in Nepal by [10]. According to the cross-sectional study, the pandemic presented more stressors to nursing students who were working in the hospital and worried about the necessity of adhering to COVID-19 precautions. In agreement, the pandemic caused anxiety and stress, according to another study by [5]. The authors conducted the study in the United States among 50 nursing students to explore anxiety and stress experienced by nursing students and identify sources of support during the transition to online learning. The study points out, anxiety and stress were evident due to a lack of PPE among nursing students who work in the hospital during the pandemic.

##### Stress from COVID-19 Infection

Four articles reported on the issue of stress from COVID-19 infection. The first study by [7], which was conducted in Israel on 244 nursing students, found that a high level of anxiety was related to a fear of getting infected by the COVID-19 virus; the anxiety score was 13.7 out of 14 according to generalized anxiety disorder 7- item scale. The second study by [16] in Croatia on 33 nursing students pointed out that 19 of the students felt stress and fear about the elderly members of their families getting infected by COVID-19. At the same time, 15 of them worried about getting infected by COVID-19 in the clinical setting.

The third study by [19] was also conducted in Turkey on 662 nursing students. A total of 68% of them worried about being infected by the COVID-19 virus, and 78.9% of the students apply adequate precautions against infection to protect themselves, 97% wash their hands frequently, 82.3% wear a mask, and 92.9% maintain a social distance. The fourth study by [10] involved 184 nursing students from universities in Nepal. The study reported that 21% of the students worried about their families being infected by the COVID-19 virus, while 8.2% worried about themselves being infected by the virus. Additionally, the studies established the coping mechanisms of the nursing students during the pandemic.

#### 3.3.2. Theme 2: Coping Strategies

Six articles identified various coping mechanisms used by nursing students. The three most prominent subthemes are addressed in the following section. The first subtheme is seeking information and consultations. In addition, developing a positive attitude has emerged as one of the most used coping strategies by nursing students. The second subtheme is staying optimistic, whereby the nursing students showed tendencies of having generalized positive expectations for the outcome. The third subtheme is getting transference by employing efforts to transfer one’s attention from stressful situations. Examples of transferring attention included eating well, exercising, and getting enough sleep. The topic assesses how distancing from the challenges associated with the virus, such as anger and frustration, has helped or can help nursing students in universities worldwide to mitigate the negative impacts of COVID-19 on their social life and enhance their academic experience.

##### Seeking Information and Consultation

Seeking information and consultation is a possible coping strategy for nursing students during the COVID-19 pandemic. The cross-sectional study conducted by [7] on 244 nursing students discovered that COVID-19 increased concerns among many nursing students, not just about this disease but also about the disruption of their daily routines, financial challenges, spending much time away from their friends and family, and the new paradigm of moving academics online with remote learning. The study further noted that maintaining a positive attitude in seeking information and consultation was a positive coping strategy associated with better mental outcomes among nursing students.

##### Staying Optimistic

The importance of staying optimistic was evident in another study in Saudi Arabia. Another author [14] conducted a cross-sectional study on 124 participants in Saudi Arabia to find 79% of nursing students in the country understood that by staying optimistic, they had a viable strategy for coping with COVID-19-related stressors, such as fear of getting infected and the deaths of patients as a result of this disease. Therefore, optimism has emerged as a positive coping strategy that urged nursing students to stabilize and gain psychological adaptation during this period.

##### Transference

Four articles emphasized the importance of transference as a coping strategy for COVID-19 by nursing students. Another author [17] conducted a study in Spain and noted the effect of the COVID-19 pandemic on nursing students’ challenging daily lifestyles. The pandemic created fear, anxiety, and stress among Spanish nursing students. Consequently, transference, such as doing regular exercise and talking with other people, positively reduced stress among nursing students. Authors [13] agreed with [17] on transference behaviors among nursing students after a descriptive cross-sectional study conducted in Egypt and Saudi Arabia. The authors noted that getting social support from peers was one of the most effective coping strategies for nursing students during the COVID-19 pandemic.

The third study was a cross-sectional study by [15], conducted on 316 nursing students in Turkey to evaluate anxiety levels and coping strategies during the pandemic. According to the authors, nursing students suffered from moderate anxiety levels as a result of COVID-19. The study further found out that 48.1% of the students used the eating coping method, and 77.8% spend time on the internet; this indicates ineffective coping strategies, which are associated with stressful events during the pandemic.

The fourth article by [20] underlined the importance of transference after conducting another study in Australia and India. The comparative study assessed anxiety levels and coping strategies among nursing students. The cross-sectional study indicated that these student nurses inevitably experience heightened anxiety. Therefore, one of the coping strategies applied by Indian nursing students to reduce stress levels is exercise and talking to other people.

## 4. Discussion

The scoping review explored the relevant evidence on the stressors and coping strategies of nursing students during the COVID-19 pandemic. The review relied on evidence from 13 studies. The inclusion of the 13 studies and subsequent critical comparison of the evidence revealed compelling stressors of the nursing students and the various mechanisms for coping with the disruption of the pandemic on the learning process.

The nursing students cited distance learning as a source of stress. The new technological-based option of delivering nursing education brings technical issues, internet problems, and poor management of online classes. Challenges arise for the nursing students because they prefer conventional learning as opposed to the online distance learning options [23]. Distance learning might not enhance more student-centered learning, monitoring, and teaching assessments than the conventional classroom does during a lockdown.

Assignments and workload emerged as compelling stressors for the nursing students as COVID-19 created a newly structured learning process. The online learning environment compels educators to provide assignments to keep up with skill development as they do in the normal classroom setting. The stressful learning circumstances may not translate into quality skills, as the students require real-life demonstrations or simulations [24]. The completion of the pre-licensure nursing students becomes difficult because they rely on the excessive workload to meet the non-direct care hours as well as optimizing virtual clinical experience with their supervisors.

The review further revealed that clinical training has created anxiety and stress among nursing students during the pandemic. The pre-licensure students are among healthcare workers without access to PPE at times, so they face the risk of contracting the virus. According to [24], the students might lack proper preparation through mentorship or preceptorship to handle the challenging active care environment with positivity rates of COVID-19 increasing every day. The fears, anxiety, and stress arise from the virus as well as students’ inability to reach a proper learning trajectory [25]. The nursing students lack proper familiarity with self-efficacy, communication, and resilience-oriented mechanisms.

The stress of clinical training underscores the impact of the COVID-19 infections without proper clinical skills, treatment mechanisms, and overall response repertoire. The review emphasized the effort of social distancing, wearing masks, and washing hands besides taking other necessary precautions against the virus. The students face micro-aggressions and a limited chance of making choices for the safety of their physical as well as psychological health as they do in the active care setting or virtual learning environment [26]. The universities or nursing colleges contribute to the challenge by allowing nursing students to enter high-risk health environments without the proper skills.

The stress and anxiety from the learning or high-risk environments have necessitated the adoption of coping mechanisms by the nursing students. The review ascertains the wide-ranging use of information and consultations to develop the right attitude during the COVID-19 pandemic. The information is critical in overcoming confinements, eliminating uncertainties, and teaching new methodologies for overcoming COVID-19 mental health challenges among learners [27]. The information and consultation of supervisors in schools or high-risk environments should eliminate uncertainty about COVID-19 as well as the completion of a nursing curriculum in readiness for registration.

Optimism emerges as the critical coping tool for nursing students hoping to complete their courses through virtual learning. The review affirms the method as a psychological adaptation to the new learning structure created by the pandemic. Optimism underlines the hopes for better eventualities for the learning environment as the nursing transitions to the professional nursing practice [28]. The findings ascertain continued efforts by nursing students to adopt behaviors that promote their well-being while hoping for a restoration of the normal learning approach.

Transference is another effective coping mechanism for nursing students who have hopeful prospects about the trajectory of their nursing education. The nursing students deal with the virtual learning environment, clinical training in a high-risk environment, and workloads with exercises or socialization. Social support is critical due to the overall life interruptions and hopeful prospects of completing nursing programs despite the COVID-19 pandemic [29]. The newly structured learning environment provides nursing students with challenging choices and has immense implications for the nursing practice.

Nursing education should adopt other impactful and interactive methods, such as video-simulated options. The nursing students could reduce fears and anxiety or stress by working with interactive modalities more than working on theoretical tasks without the capacity to enhance their practical nursing skills. On the other hand, continuous counseling and social support are essential in the communities, as universities and colleges seek better engagement for the pre-licensure nursing students. The strategies will complement the coping mechanisms of transference, seeking information or consultation, and optimism because nursing students anticipate a smooth transition to the practice during the COVID-19 pandemic.

However, the scoping review had several limitations. The review pointed towards available studies in stressors and coping mechanisms of nursing students during COVID-19 that opposite the research that allows searching of new findings. The method further lacked the articulation of the risk of bias that reduced the reliability of the outcomes. The results are based on the relatively novel COVID-19 topic, which continues to evolve over time, and thus, the overall reliability remains contentious.

COVID-19 painted a grim picture of the world’s lack of preparedness for a pandemic such as this. In this review about stressors and coping strategies, it was discovered that nursing students suffered from stressors during this pandemic in their academic journeys. There are various stressors that nursing students face, including stress from distance learning, stress from assignments and workloads, stress from clinical training, and stress from COVID-19 infection. In response to these stressors, nursing students have developed coping strategies that were employed to adjust to these COVID-19-related stressors, such as seeking information and consultation, staying optimistic, and transference.

## 5. Recommendation

The review contends that using different mechanisms improves the quality of nursing education delivered to the nursing students during the pandemic. The coping mechanisms discussed in the study imply the importance of creating a structured learning environment to enhance the outcomes of the nursing students while minimizing their susceptibility to anxiety and stress. Further studies will suffice to deliberate on mental issues and solutions facing nursing students during the COVID-19 pandemic.

## Figures and Tables

**Table 1:** PICOT framework.

PICOT	Content	Question
P	Nursing students	What are the stressors and coping strategies during the COVID-19 pandemic among nursing students?
I	Not applicable	
C	Not applicable	
O	Stressors and coping strategies	
T	During the COVID-19 pandemic	

**Table 2:** Quality-approved total score allocation. Maximum score = 36.

Author (s)	1 Abstract/Title	2 Introduction/Aims	3 Method/Data	4 Sampling	5 Data Analysis	6 Ethics/Bias	7 Results	8 Transferability	9 Implications	Total
Deo et al. (2020) [10]	4	3	3	4	4	3	4	3	2	30
Begam and Devie (2020) [11]	4	3	3	1	3	3	3	3	1	24
Masha’al et al. (2020) [12]	4	3	4	4	4	3	4	3	3	32
Fitzgerald and Konrad (2021) [5]	4	3	4	4	4	4	4	4	4	35
Hussien et al. (2020) [13]	4	4	4	4	4	4	4	3	4	35
Begum (2020) [14]	4	2	2	1	2	4	4	3	1	23
Savitsky et al. (2020) [7]	3	3	4	4	4	3	4	3	2	30
Zeynep (2020) [15]	4	3	4	4	4	3	4	3	1	30
Lovrić et al. (2020) [16]	4	3	4	4	4	3	3	3	4	32
Gallego-Gomez et al. (2020) [17]	3	3	4	4	4	3	4	2	1	28
Subedi et al. (2020) [18]	4	3	4	4	4	3	3	2	2	29
Aslan and Pekince (2020) [19]	3	2	4	3	4	4	4	3	4	31
Kochuvilayil et al. (2020) [20]	4	3	4	4	4	4	3	3	3	32

**Table 3:** Characteristics of the reviewed studies.

Author (s)	Sample Size and Setting	Country of Study	Study Design	Measurement Tool	Main Findings
Deo et al. (2020) [10]	148 nursing students at Nobel college	Nepal	Cross-sectional, survey-based study	A digitalized structured questionnaire contained a total of 45 questions to assesssocio-demographic charcteristics, associative factors, DASS (Depression, anxiety, stress scale) and ISI (Insomnia Index Scale).	The study found out that the COVID-19 pandemic presents significant effects on nursing students in Nepal. Some of the COVID-19-related stressors among this population include the fear of delayed graduation.
Begam and Devi e (2020) [11]	244 nursing students in an online mode in three schools of nursing, Assam	India	Cross-sectional study	Google Form that contained Tool I for collecting sociodemographic data and Tool II for the Perceived Stress Scale (10) by Cohen Sheldon with 5-Point Likert Scale	The study found out that they had experienced moderate levels of stress due to COVID-19.
Masha’al et al. (2020) [12]	335 nursing students in an online platform through Jordan universities	Jordan	Mixed methods	Online survey in Google Forms that contained the students’ sociodemographic characteristics and the Higher Education Stress Inventory	According to this study, COVID-19 presents particularly stressful experiences for nursing students doing distance learning.
Fitzgerald and Konrad (2021) [5]	50 nursing students participating in a web-based platform	USA	Descriptive study	Web-based survey developed through Qualtrics Software to develop a checklist based on a 10-item anxiety Symptoms Checklist	The study sought to unearth the stress and anxiety experienced by nursing students during COVID-19, nursing students feeling anxious and overwhelmed from handling the academic workload and stress from a lack of PPE in the workplace.
Hussien et al. (2020) [13]	284 nursing students at the Faculty of Nursing, Zagazig University, Egypt, Faculty of Applied Medical Science, Taibah University, and Al-Ghad International Colleges, KSA	Saudi Arabia and Egypt	Descriptive cross-sectional design	Questionnaire with a sociodemographic datasheet, the Emotional Intelligence Scale, and the Intolerance of Uncertainty Scale	Hussein et al. (2020) study found out that emotional intelligence is an important coping strategy for nursing students in these two countries during the COVID-19 pandemic. Comparing the two sets of students, Saudi nursing students demonstrated higher levels of emotional intelligence than their Egyptian counterparts.
Begum (2020) [14]	124 nursing students participating in online research during lockdown	Saudi Arabia	Quantitative cross-sectional study	Adapted questionnaire from a Chinese study that detailed demographic variables of age and gender, and 15 knowledge-based, 10 attitude-based, and 5 practice-based questions	According to this study, Saudi nursing students have a satisfactory level of knowledge about COVID-19. In addition, these students also possessed a positive attitude towards the pandemic and the possibility of overcoming it.
Savitsky et al. (2020) [7]	244 nursing students at a nursing department during a national lockdown	Israel	Cross-sectional study	Generalized Anxiety Disorder 7-Item Scale that outlined a cut-off point of 10 for moderate anxiety and of 15 for severe anxiety levels	The study found that the most common coping mechanisms among nursing students during the pandemic were resilience, seeking information, mental disengagement, humor, and the use of spiritual support.
Zeynep (2020) [15]	316 nursing students at a university in the Eastern Black Sea region, Turkey	Turkey	Cross-sectional study	Personal information form Generalized Anxiety Disorder-7 Scale Stress Coping Strategies Scale	The COVID-19 pandemic has affected the overall performance of nursing students. Nevertheless, the study found that the participants were demonstrating moderate levels of anxiety.
Lovrić et al. (2020) [16]	33 nursing students at the Faculty of Dental Medicine and Health, Osijek	Croatia	Qualitative study	Online form with two major questions	All students were aware and concerned about the issues of misinformation on social media and the risky behavior of the population. Additionally, most of them were worried about getting infected and were concerned about their families’ well-beings. Therefore, they constantly applied protective measures. Moreover, the students understood their responsibility to the community and the importance and risks of the nursing profession. They also described negative experiences with public transportation and residing in the student dorm.
Gallego-Gomez et al. (2020) [17]	142 students at the Faculty of Nursing of the Catholic University of Murcia (UCAM) located in Murcia, Spain	Spain	Observational	Student Stress Inventory–Stress Manifestations (SSI–SM) questionnaire with 19 items in a 5-point Likert-type score	The nursing students experienced an increase in stress levels during the lockdown. They also experienced family and financial problems during this period. Their main coping strategy was engaging in physical exercise.
Subedi et al. (2020) [18]	1116 nursing students at different nursing colleges in Nepal	Nepal	Descriptive cross-sectional online survey	Self-administered questionnaire in an online survey	Close to half of the teachers (42.3%) indicated that they witnessed disturbances to their online classes due to electricity issues. Moreover, 48.1% of them stated that they had challenges with internet access. Over half of the students polled (63.2%) stated that their online learning was affected by electricity and 63.6% had internet problems; only 64.4% of the students had internet access for their online classes.
Aslan and Pekince (2020) [19]	662 nursing students at Inonu, Kilis, and Bingol Universities	Turkey	Cross-sectional design	Information form and perceived stress scale	Stress was prevalent among many nursing students during the COVID-19 pandemic. Nursing students between the ages of 18 and 20 years and female students reported higher levels of stress. The study also found out that the most important stressors among these students included watching the news, worrying about the risk of infection, and the imposed curfew.
Kochuvilayil et al. (2020) [20]	99 Australian and 113 Indian nursing students at NSW and Kerala	Australia and India	Cross-sectional study a comparative study	Online survey prepared through Survey Monkey	Student nurses inevitably experience heightened anxiety.

## Data Availability

No new data were created or analyzed in this study. Data sharing is not applicable to this article.
